# The regulation of autophagy by the miR-199a-5p/p62 axis was a potential mechanism of small cell lung cancer cisplatin resistance

**DOI:** 10.1186/s12935-022-02505-1

**Published:** 2022-03-15

**Authors:** Tiezhi Li, Helin Zhang, Zhichao Wang, Shaolin Gao, Xu Zhang, Haiyong Zhu, Na Wang, Honglin Li

**Affiliations:** 1 Department of Thoracic Surgery, The Second Hospital of Hebei Medical University, Shijiazhuang, China; 2 Department of Pediatrics, The First Hospital of Hebei Medical University, Shijiazhuang, China; 3 Department of Respiratory and Critical Care Medicine, The Second Hospital of Hebei Medical University, Shijiazhuang, China

**Keywords:** MDR, H446, Autophagy, MiR-199a-5p, Cisplatin, p62

## Abstract

**Background:**

Autophagy has been found to be involved in the multidrug resistance (MDR) of cancers, but whether it is associated with resistance of small cell lung cancer (SCLC) has not been studied. Here, we hypothesized that a potential autophagy-regulating miRNA, miR-199a-5p, regulated cisplatin-resistant SCLC.

**Methods:**

We validated the MDR of H446/EP using CCK-8 and LDH. We tested the binding of miR-199a-5p to p62 using the Dual-Luciferase assay and validated the association of miR-199a-5p and p62 in SCLC samples. We overexpressed (OE) and knocked down (KD) miR-199a-5p in H446 and H446/EP and determined the expression of miR-199a-5p, autophagy-related proteins, and the formation of autophagolysosomes using QPCR, western blotting, and MDC staining respectively. These results were validated in an orthotopic H446 mouse model of SCLC.

**Results:**

H446/EP was resistant to cisplatin, etoposide, paclitexal, epirubicin, irinotecan, and vinorelbine. Exposure of cisplatin at 5 μg/ml for 24 h increased LC3II/LC3I, ATG5, p62, and the formation of autophagolysosomes in H446 cells, but not in H446/EP cells. The expression of miR-199a-5p was up-regulated in H446/EP compared to H446. MiR-199a-5p directly targeted the p62 gene. The expression of miR-199a-5p and p62 were correlated in SCLC samples. In H446 and H69PR, the OE of miR-199a-5p increased LC3II/LC3I, p62, and the formation of autophagolysosomes, but not ATG5, while the KD of miR-199a-5p decreased p62, but did not affect LC3II/LC3I, ATG5, and the formation of autophagolysosomes. In H446/EP, the OE of miR-199a-5p decreased p62 only. These results were generally consistent to results in the animal tumor samples.

**Conclusions:**

The regulation of autophagy by the miR-199a-5p/p62 axis was a potential mechanism of small cell lung cancer cisplatin resistance.

**Supplementary Information:**

The online version contains supplementary material available at 10.1186/s12935-022-02505-1.

## Background

More than 80% of clinical lung cancer cases are diagnosed as non-small cell lung cancers (NSCLC) and only less than 20% as small cell lung cancer (SCLC) [1, 2], but NSCLC typically grow at a slower rate than SCLC and are difficult to be discovered until they have advanced [3]. As SCLC can be diagnosed at earlier stages, chemotherapy is the major treatment for SCLC instead of surgical treatment, resulting in more drug resistance issues [4]. Multidrug resistance (MDR) is an innate and/or acquired ability of cancer cells to survive against a wide range of chemotherapy drugs [5]. In clinical cancer chemotherapy, MDR has been one of the tough dilemmas. Over 90% mortality of cancer patients is associated with MDR. To date, a variety of mechanisms has been proposed to be involved in the MDR of cancer cells during chemotherapy, including enhanced efflux of drugs, genetic factors (gene mutations, amplifications, and epigenetic alterations), growth factors, increased DNA repair capacity, and elevated metabolism of xenobiotics [6]. These mechanisms reduced the therapeutic efficacy of chemotherapy, resulting in the insensitivity of cancer cells to the treatment. Although research has revealed many potential mechanisms underlying MDR, the understanding of MDR is still lacking and no effective way has been found to solve the problem of MDR ideally [7].

Autophagy, as a type II programmed cell death, plays crucial roles with autophagy-related (ATG) proteins in cancer [8]. Autophagy has been suggested to be one of the factors that might affect MDR [9–11]. It is characterized by a self-digestion pathway that activates lysosomes to degrade damaged or superfluous cell components in the cells [12, 13]. Studies have shown that autophagy prevents cells from apoptosis, hypoxia, and damage stress responses. As a complex cell behavior, autophagy involves many biological processes and might interfere with MDR pathways [14, 15]. Light chain 3 (LC3) is a central protein in the autophagy pathway where it functions in autophagy substrate selection and autophagosome biogenesis, which is the most widely used biomarker of autophagosomes [16]. Another widely accepted autophagy biomolecule is p62, which is also named sequestosome 1 (SQSTM1) [17]. The p62 protein is degraded by autophagy and accumulates when the autophagy level is reduced [18]. However, p62 is a multi-functional protein in autophagy. The binding of p62 directly to LC3 via a short LC3 interaction region is one of the most critical mechanisms to deliver selective autophagic cargo for the bioprocess [19]. The p62 overexpression increases the aggregation of ubiquitinated proteins and has a protective effect on cell survival, while p62 decrease results in some diseases by damaging autophagic degradation [20].

A microRNA (miRNA) miR-199a-5p has been found closely related to autophagy and drug resistance [21]. This miRNA plays roles in multiple cancers, including lung cancer [22–24], laryngeal cancer [25], colorectal cancer [26], etc. A previous study has found that miR-199a-5p inhibited protective autophagy and reversed chemoresistance by regulating DRAM1 protein in leukemia cells [27, 28]. Therefore, we proposed that miR-199a-5p might mediate the effect of cisplatin on the autophagy of lung cancer cells. In the present study, we explored the correlation between autophagy and MDR development in SCLC H446 cells and investigated the role of miR-199a-5p. Our data revealed the role of miR-199a-5p in the autophagy regulation and cisplatin resistance in SCLC.

## Methods

### Cell lines and cell culture

Small cell lung cancer cell (SCLC) line NCI-H446 [H446] (ATCC^®^ HTB-171™) and H69PR (ATCC^®^CRL-11350™) were purchased from ATCC (Washington, USA). The cell lines were cultured using the RPMI‑1640 medium (Gibco; Thermo Fisher Scientific, Inc., Waltham, MA, USA) with 10% FBS (Gibco; Thermo Fisher Scientific, Inc., Waltham, MA, USA) in a 37 °C 5% CO_2_ incubator. The multidrug resistance H446 sub-cell line H446/EP was developed from H446 with increasing concentration selection of etoposide (Sigma-Aldrich, St. Louis, MO, USA) combined with cisplatin (Sigma-Aldrich, St. Louis, MO, USA), both increased from 50 ng/ml to a final dose of 1000 ng/ml. The H446/EP obtained were cultured in the drug-free medium for over 10 generations before the experiments [29].

### IC50 of viability and EC50 of cell damage

IC50 of drugs was determined using the Cell Counting Kit-8 (CCK-8, Sigma-Aldrich, St. Louis, MO, USA) assay and LDH assay as previous studies [30, 31]. Briefly, cells were cultured in 96-well plates with drugs accordingly. At the endpoint of the exposure, the CCK-8 reagent (10 ml/well) was added. After 3 h of incubation at 37 °C, the absorbance at 450 nm was evaluated using a microplate reader (Bio-Rad, Model 680). The cisplatin, etoposide, paclitexal, epirubicin, irinotecan, and vinorelbine were purchased from Sigma-Aldrich (St. Louis, MO, USA). Then the IC50 of viability was calculated. In the LDH assay, the cells were plated in 96-well plates (3–5 × 10^3^/well) for 12 h. LDH in the medium was then detected using the Cytotoxicity Detection Kit (Sigma-Aldrich, St. Louis, MO, USA). LDH assays were carried out according to the manufacturer’s instructions. The absorbance was measured at 490 nm using the a microplate reader. The LDH level in cells treated with Triton X-100 was used as positive control and to normalize the results. Then the EC50 of cell damage was calculated.

### Cell transformation

The overexpression (OE) and knockdown (KD) of miR-199a-5p were achieved by transformation of sh-miR-199a-5p vector or miR-199a-5p expression vector into cells respectively. Briefly, the miR-199a-5p or its shRNA coding sequence was cloned into the pLV-mCherry (Plasmid #36084) vectors. Negative expression control vectors (OENC) and shRNA control vectors (KDNC) were also constructed with the same vector. Lipofectamine^®^ 2000 was used to transform the cells (5 mg DNA/10 mm cell culture dish). The transformation was validated by observing the RFP marker in the transformed cells. The vectors were purchased and constructed by Beyotime Biotechnology (Shanghai, China).

### Western blotting assay

The protein expressions were analyzed using the western blotting assay as described previously [32]. Briefly, cells were lysed in the RIPA buffer (Sigma-Aldrich, St. Louis, MO, USA) plus protease inhibitor (Pierce Protease Inhibitor Mini Tablets, Thermo Fisher Scientific, Inc., Waltham, MA, USA). SDS-polyacrylamide gel electrophoresis (PAGE, Sigma-Aldrich, St. Louis, MO, USA) electrophoresis was used to separate the proteins in samples. Then the proteins were transferred to 0.2-μm polyvinylidene difluoride membranes (Thermo Fisher Scientific, Inc., Waltham, MA, USA). The membranes were blocked with blocking buffer (Pierce™ Protein-Free Blocking Buffer, Thermo Fisher Scientific, Inc., Waltham, MA, USA) for 1 h. The membranes were then incubated with primary antibodies at 4 °C, overnight, and then secondary antibodies at room temperature for 2 h (dilution following the recommended concentration of the antibody respectively). The ECL Detection Reagent (Sigma-Aldrich, St. Louis, MO, USA) was used to visualize the target protein. The primary antibodies used in this study were as follows: LC3B (1:800, 2775S, Cell Signaling); P62/SQSTM1 (1:2500, 18420-1-AP, Protein Tech); ATG5 (1:1000, GTX113309, GeneTex), and β-Actin (1:5000, sc-1615) (Santa Cruz Biotechnology, Dallas, TX, USA). All the secondary antibodies were purchased from the Abcam (Cambridge, UK).

### Autophagolysosomes observation

Monodansylcadaverine (MDC, Sigma-Aldrich, St. Louis, MO, USA) staining was used to observe autophagolysosomes as described previously [33]. Briefly, cells were cultured in a 6-well plate under testing conditions. At the endpoint of the exposure, the cells were incubated with MDC (50 µmol/l) and PureBlu™ DAPI Nuclear Staining Dye (#1351303) for 30 min at 37 °C. Then the cells were washed with precooling phosphate-buffered saline (Sigma-Aldrich, St. Louis, MO, USA), followed by the observation using a fluorescence microscope (GXM UltraDIGI-SBMF1, USA). The signals were quantified using ImageJ software.

### Real-time quantitative PCR

The expression of miR-199a-5p was evaluated using real-time quantitative PCR (QPCR) as described previously [34]. Briefly, TRIzol reagent (Vazyme) was used to extract total RNA from cells following the manufacturer’s instructions. The target RNAs were reverse-transcribed to cDNA using the TaqMan MicroRNA Reverse Transcription Kit (Applied Biosystems). Real-time PCR analysis was performed using KiCqStart^®^ SYBR^®^ Green qPCR ReadyMix™ (Sigma-Aldrich, St. Louis, MO, USA) with a Real-Time PCR platform (CFX96, BIO-RAD). All the PCR primers used in the study were synthesized by Thermo Fisher Scientific, Inc. (Waltham, MA, USA). The expression was normalized to RNU6-1 miRNA expression using the ^△△^CT method.

### Dual-Luciferase Reporter Assay

Firefly/Renilla Dual Luciferase Assay (Sigma-Aldrich, St. Louis, MO, USA) was used to test the binding of miR-199a-5p to wild-type (WT) or mutated coding sequence of p62 as previously described [35]. Briefly, cells were plated in a 96-well plate and negative plasmids or reporter plasmids with WT or mutated p62 sequence were transformed to cells. The sequences were shown in Fig. 2C. After 48 h transformation, cells were lysed, and the luciferase signal was measured following the protocol with the microplate reader (GXM UltraDIGI-SBMF1, USA).

### Tissue collection

Cancer tissues were collected from 30 patients with SCLC surgical treatment or biopsy from the Second Hospital of Hebei Medical University. Patients’ information was listed in Additional file 1. Samples were fixed, embedded in paraffin, and stored in 4 °C. All donors were over 18 years old and have given formal consent to the use of their samples. The study has been approved by the Ethics Committee of the First Hospital of Hebei Medical University.

### Immunochemistry staining

P62 (SQSTM1) staining was done by immunochemistry using SQSTM1Antibody (SQSTM1/p62 Antibody #5114). Briefly, paraffin-embedded tissue samples were deparaffinized in xylene, rehydrated through graded ethanols, and then submerged into the citric acid buffer for heat-induced antigenic retrieval, blocked with 10% bovine serum albumin, incubated with SQSTM1 primary antibodies at 4 °C overnight, and developed using the DAKO ChemMate Envision Kit HRP (Dako-Cytomation, Carpinteria, CA, USA) followed by counterstaining with hematoxylin, dehydration, clearing and mounting.

### Animal experiments

This study was performed in accordance with a protocol approved by the local Ethics Committee of Animal Experiments. Male athymic nude mice were purchased from the local Animal Center and the mice were housed and maintained in conditions in facilities approved by the local Ethics Committee of Animal Experiments. Mice 8 to 10 weeks old were used for model establishment. The model was established as described previously [36]. Mice were anesthetized with sodium pentobarbital (50 mg/kg). A small skin incision to the left chest wall was made approximately 5 mm to the tail side of the scapula. The cells were suspended at 2 × 10^6^ cells/ml in Hanks’ balanced salt solution (HBSS; Sigma Chemicals Co., St. Louis, MO) and 0.5 mg/ml solution of growth factor-reduced Matrigel. 75 μl of cells mix were injected into the left lung. H446 and H446/EP cells were used for the model. Mice were treated intraperitoneally (i.p.) with cisplatin PBS solution at 3 mg/kg every 3 days from day 3 to the end of the experiment. 40 mice were injected with H446 and the other 40 mice were injected with H446/EP. Among 40 mice injected with H446, 20 mice were treated with cisplatin, the other was treated with vehicle, the same setting for H446/EP. For each group, half of the mice (n = 10), were killed at day 40, and tumors were collected, while the others were treated until death, and the survival days were recorded.

### Statistical analysis

Data are presented as means ± SD. Student’s t-test or one-way ANOVA analysis was used to analyze significance. A p-value of 0.01 or lower was considered significant.

## Results

### Cisplatin induced autophagy in H446 but not H446/EP

**Fig. 1:**
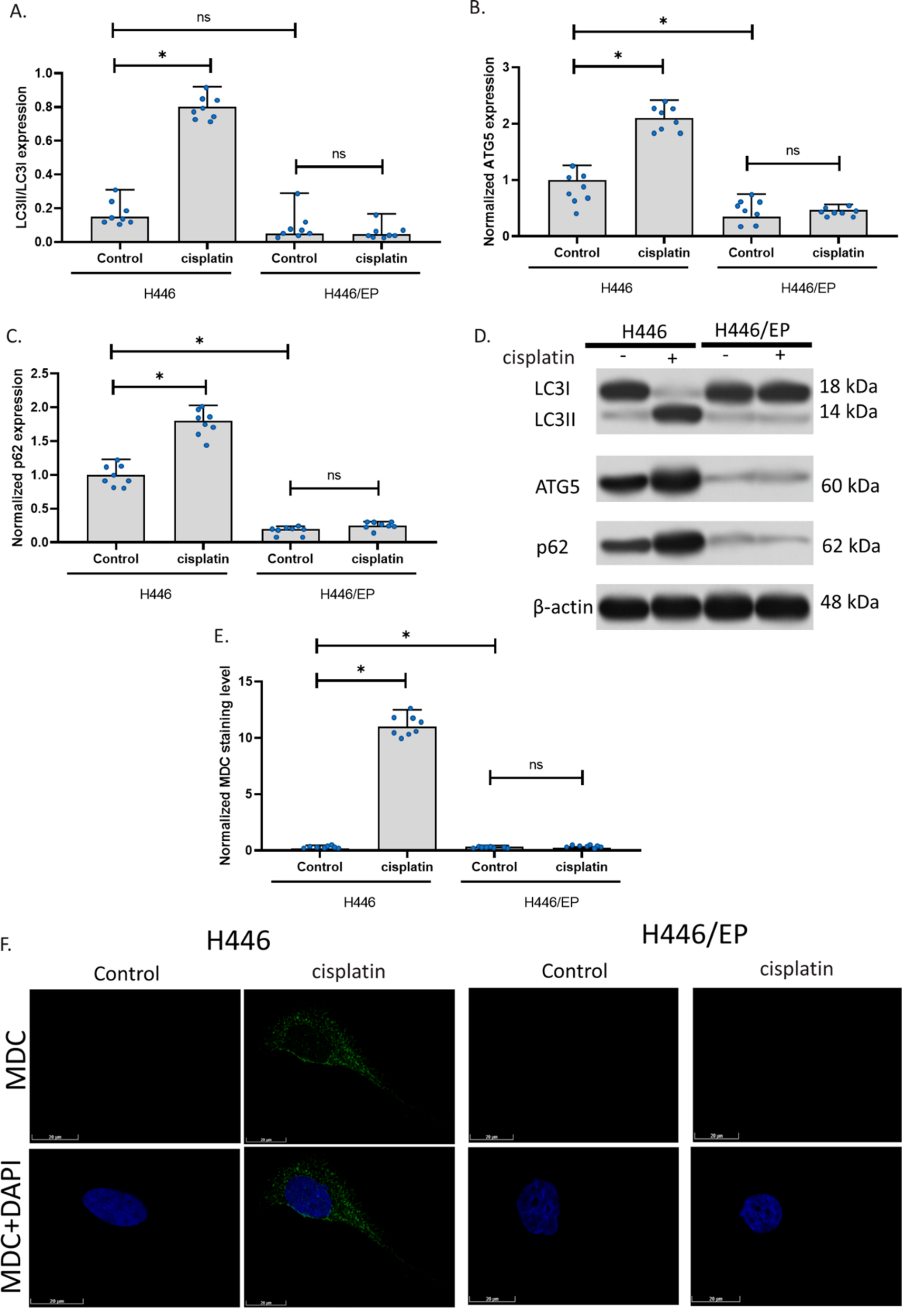
The difference in autophagy in H446 and H446/EP. **A**–**C** LC3II/LC3I, ATG5, and p62 expressions in H446 and H446/EP. The expression was measured using the western blotting assay. **D** Representative images of **A**–**C**. **E** MDC staining of autophagolysosomes in cells. **F** Image of autophagolysosomes in cells with drug exposure. The fluorescence images of autophagolysosomes were captured after the MDC and DAPI staining of cells. (*p < 0.01)

Firstly, we generated MDR H446 cells and validated their resistance using CCK assay and LDH assay, as shown in Additional file 1: Tables S1, S2. Based on the IC50 of viability and EC50 of cell damage, we used an exposure of cisplatin at 5 μg/ml for 24 h in the subsequent study. To test the hypothesis that autophagy is involved in the resistance of H446 cells, we determined three indicators for autophagy including the ratio of LC3II and LC3I expression, the level of ATG5, and the levels of p62. Results showed that cisplatin increased LC3II/LC3I, ATG5, and p62 in H446 cells, but not in H446/EP cells. This indicated that the drug resistance of cisplatin was resulted (at least partly) from the insensitivity of autophagy induction. In addition, compared to H446, H446/EP had a similar ratio of LC3II/LC3I, but a significantly lower level of ATG5 and p62. This suggested that after a long time of exposure to cisplatin, H446 might develop autophagy-associated MDR mechanisms (Fig. 1A–D). To observe the cell activity of autophagy in the cells, we stained the autophagolysosomes with MDC. We found that autophagolysosomes were significantly increased in H446 after 24-h exposure to cisplatin. However, autophagolysosomes were not significantly increased in H446/EP after 24-h exposure to cisplatin (Fig. 1E, F). This further confirmed that cisplatin-induced autophagy was altered in H446/EP.

### MiR-199a-5p was upregulated in H446/EP and directly targeted the p62 gene

**Fig. 2:**
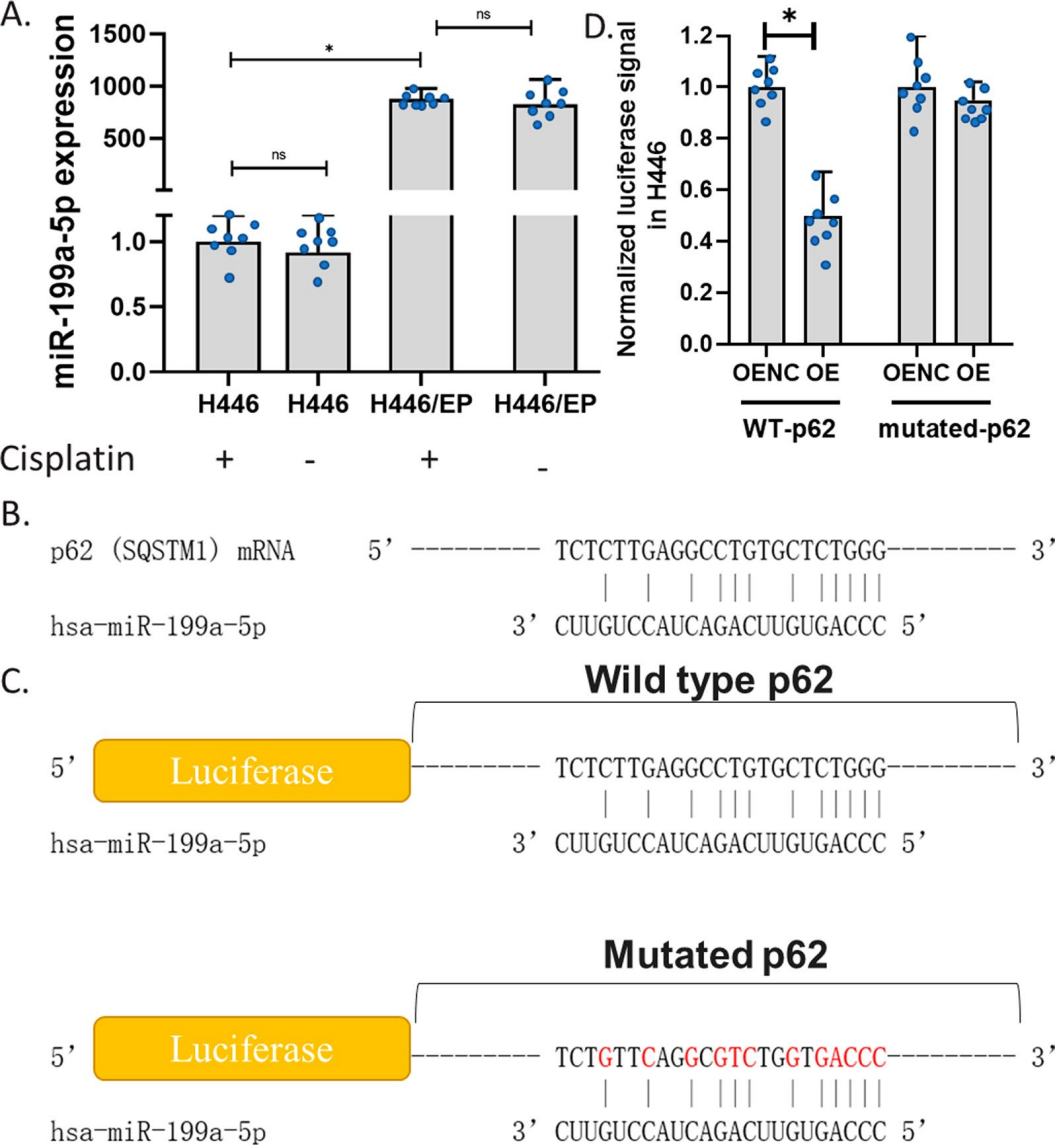
The binding of miR-199a-5p to p62 mRNA. **A** The effect of cisplatin on miR-199a-5p expression in H446 and H446/EP. miR-199a-5p expression was determined using the QPCR assay. **B** The predicted binding site of miR-199a-5p to p62 mRNA. **C** Luciferase reporter gene sequence with the alignment of the miR-199a-5p gene at the predicted binding site in Dual-Luciferase Reporter Assay. **D** Effects of miR-199a-5p on luciferase signal in H446. H446 cells were co-transformed with miR-199a-5p expression vectors and wild-type (WT)-p62 or mutated-p62 vectors. The luciferase signal was determined 24 h after the transformation followed by the addition of substrate. (*p < 0.01)

To test whether it was also associated with the drug resistance in H446, we compared the expression level of it in H446 and H446/EP. Results showed that the drug resistance selection increased the level of miR-199a-5p in H446 up to 1000 times (Fig. 2A). Such a remarkable increase in miR-199a-5p expression in H446/EP suggested that miR-199a-5p might play a potential role in the drug resistance of H446. We also exposed H446 or H446/EP to 5 μg/ml cisplatin. Both of the two cell lines were not changed in the level of miR-199a-5p (Fig. 2A).

Our western blotting results have shown that the expression of p62 was down-regulated in H446/EP compared to H446, while the expression of miR-199a-5p was up-regulated in H446/EP compared to H446. Hence, we proposed that miR-199a-5p might target the p62 gene coding sequence directly. Therefore, we invested the sequence of p62 mRNA and miR-199a-5p and predicted a potential binding site. We downloaded the RNA sequence of p62 mRNA and miR-199a-5p from the NCBI, and used the Clustal Omega Sequence Alignment tools to paired the p62 mRNA sequence and reverse the complementary sequence of miR-199a-5p. Results showed that miR-199a-5p might potentially bind to p62 mRNA with 12 complementary base pairs, including 10 C-G base pairings and 5 consecutive base pairings (Fig. 2C). To test this binding, we conducted the Dual-Luciferase Reporter Assay in H446 to validate the predicted binding site. The Luciferase Reporter gene was cloned with a wild-type p62 or a p62 with mutations at the predicted site (Fig. 2D). Results showed that the overexpression of miR-199a-5p reduced the luciferase signal of samples from cells with wild-type p62 coding sequence, but it failed to affect the luciferase signal of samples from cells with mutations at the predicted site (Fig. 2B). This indicated that the miR-199a-5p only bond to wild-type p62 mRNA but not to mutated p62 mRNA. This experiment validated the direct binding of miR-199a-5p to p62 mRNA. We suggested that this binding leads to the subsequent degradation of the p62 mRNA, which is the mechanism for miR-199a-5p down-regulating p62 expression in H446 cells.

### Validation of association of miR-199a-5p and p62 in SCLC tissues

**Fig. 3:**
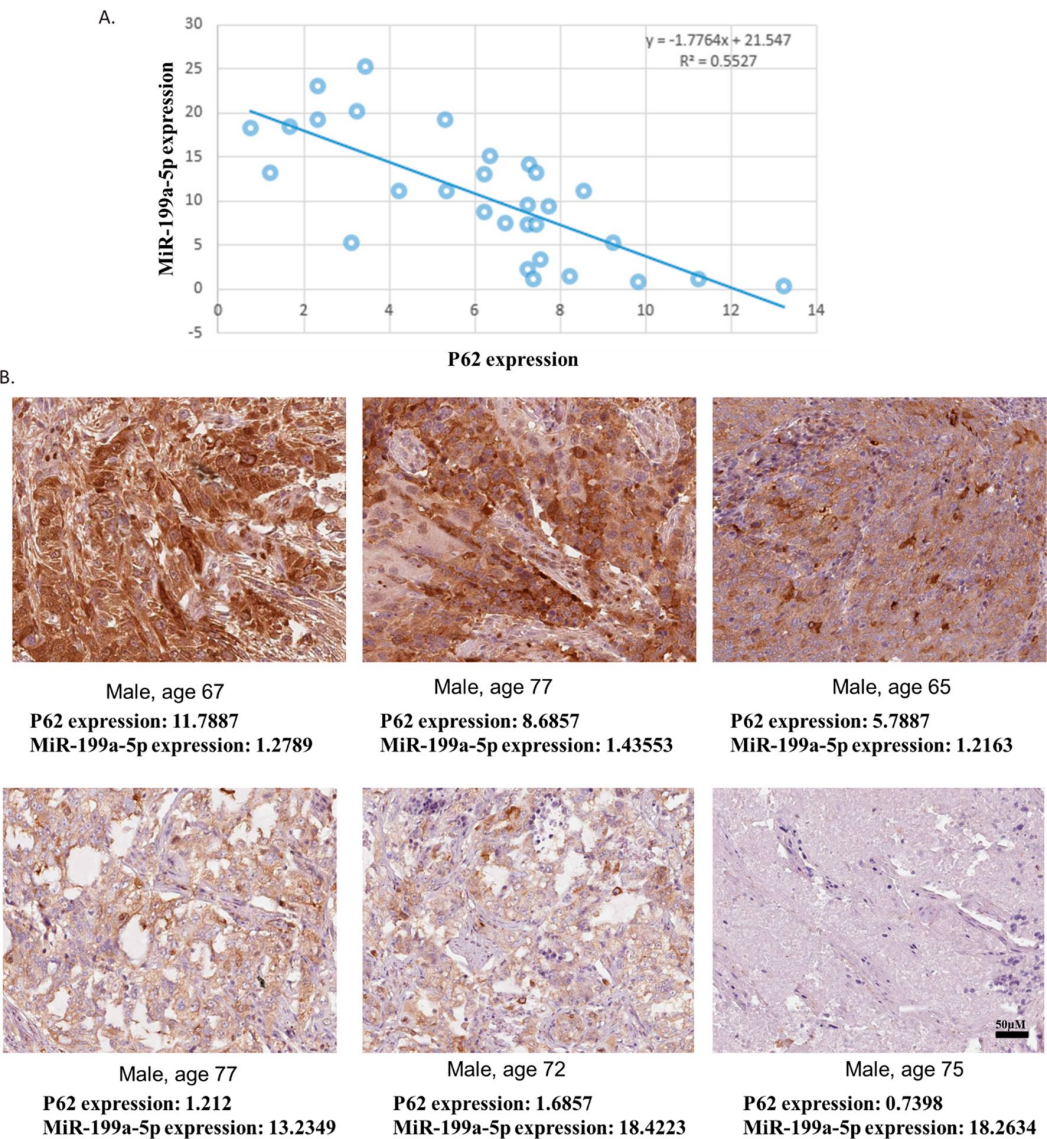
The expression of p62 and miR-199a-5p in human SCLC samples. Cancer tissues were collected from 30 patients with SCLC surgical treatment or biopsy. The expression of p62 in SCLC samples was measured using the western blotting assay. The expression of miR-199a-5p in SCLC samples was determined using the QPCR assay. **A** The correlation of p62 and miR-199a-5p expression in SCLC samples. **B** Representative images of p62 protein staining and corresponding miR-199a-5p expression

To further validate the association of miR-199a-5p and p62 in SCLC, we collected SCLC cancer tissues from 30 patients with SCLC surgical treatment or biopsy. The expression of p62 in SCLC samples was measured using the western blotting assay and the expression of miR-199a-5p in SCLC samples was determined using the QPCR assay. Subsequently, the correlation of p62 and miR-199a-5p expression in SCLC samples was calculated. Results showed that the expression of p62 was negatively correlated with the miR-199a-5p level (Fig. 3A). In the tissue staining, we found that samples with lower levels of miR-199a-5p had stronger signals of p62 protein, while the samples with higher levels of miR-199a-5p showed weaker signals of p62 protein (Fig. 3B). This further suggested that p62 expression might be negatively regulated by miR-199a-5p.

### The knockdown and overexpression of miR-199a-5p in H446

**Fig. 4:**
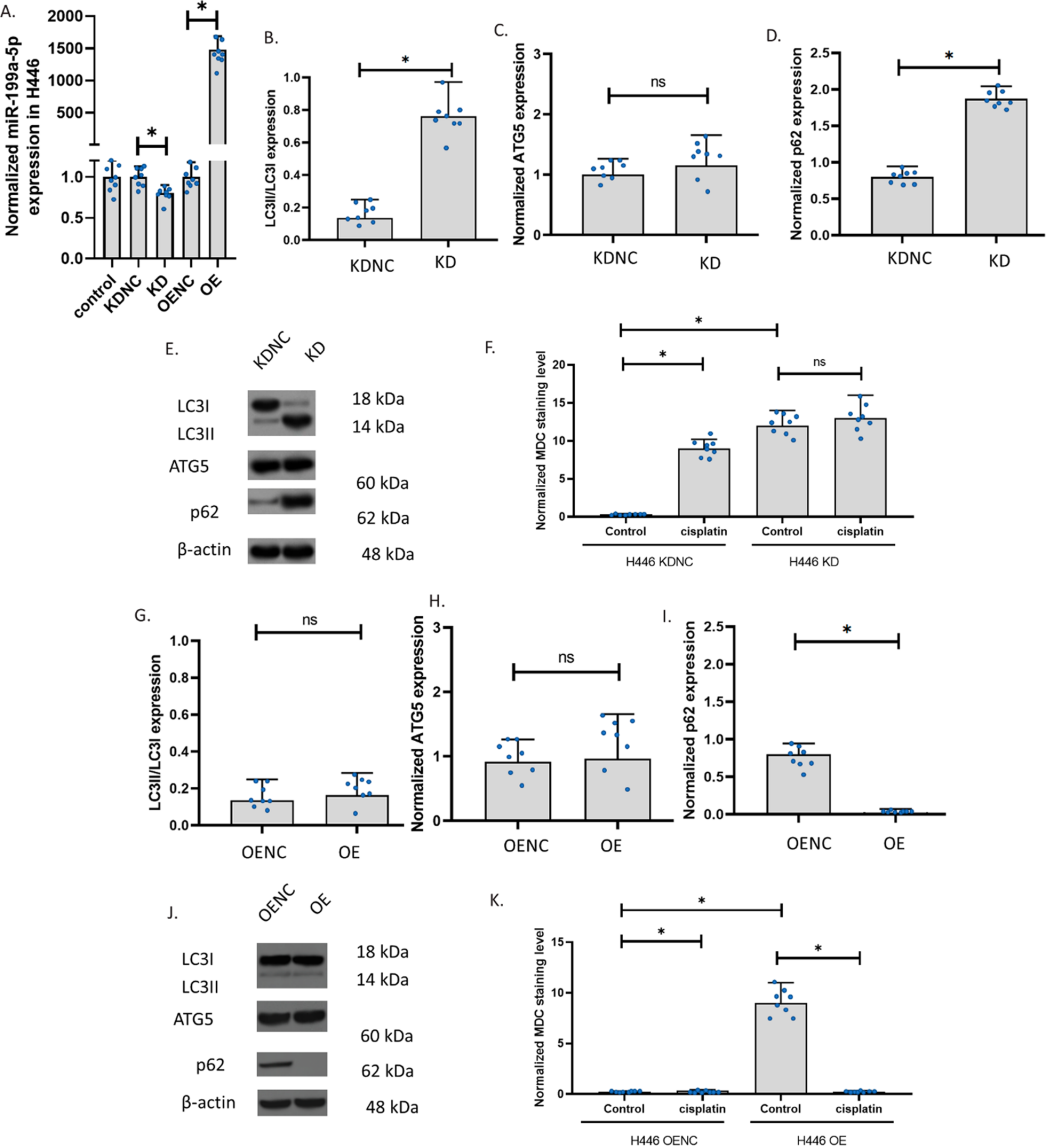
The knockdown and overexpression of miR-199a-5p in H446. **A** The effect of cisplatin and β-elemene on miR-199a-5p expression in H446 with knockdown or overexpression of miR-199a-5p. MiR-199a-5p expression was determined using the QPCR assay. Overexpression (OE); knockdown (KD); overexpression negative control; knockdown negative control (KDNC). **B**–**D** LC3II/LC3I, ATG5, and p62 expressions in H446 with knockdown of miR-199a-5p. **E** Representative images of **B**–**D**. The expression was measured using the western blotting assay. **F** The MDC staining of autophagolysosomes in cells with drug exposure. The fluorescence images of autophagolysosomes were captured after the MDC staining of cells. **G**–**I** LC3II/LC3I, ATG5, and p62 expressions in H446 with overexpression of miR-199a-5p. **J** Representative images of **B**–**D**. **K** The MDC staining of autophagolysosomes in cells with drug exposure. (*p < 0.01)

To explore the role of miR-199a-5p, we knocked down (KD) and overexpressed (OE) miR-199a-5p in H446. In H466, the knockdown slightly decreased miR-199a-5p but the overexpression increased miR-199a-5p up to 1500 folds (Fig. 4A). We determined the ratio of LC3II and LC3I expression, the level of ATG5, and the levels of p62 to measure the autophagy in H446 cells. Results showed that miR-199a-5p knockdown increased LC3II/LC3I and p62 in H446 cells, but ATG5 was not affected (Fig. 4B–E). We also observed autophagy in the cells. The staining of the autophagolysosomes with MDC in H446 cells showed that the autophagolysosomes were significantly increased in H446 with the miR-199a-5p knockdown. The autophagolysosomes were increased only in the knockdown control but not the H446 with miR-199a-5p knockdown after 24-h exposure to cisplatin. These results indicated that the miR-199a-5p might decrease p62 and the transformation of LC3I to LC3II. The decrease in miR-199a-5p can result in the insensitivity of H446 cells to cisplatin. To further explore the role of miR-199a-5p in H446, we overexpressed miR-199a-5p in H446 cells. Results revealed that, after miR-199a-5p overexpression, H446 showed a similar ratio of LC3II/LC3I and a similar level of ATG5. However, the expression of p62 was remarkably reduced (Fig. 4G–J). We suggested that the high level of miR-199a-5p decreased p62 only, but the low level of miR-199a-5p increased p62 thereby promoting the transformation of LC3I to LC3II. The staining of the autophagolysosomes with MDC in H446 cells showed that both the control and the miR-199a-5p overexpression H446 had very low signals of autophagolysosomes, the 24-h cisplatin exposure increased the signals of autophagolysosomes in control only (Fig. 4K). The overexpression of miR-199a-5p unsensitized the H446 to cisplatin.

### The knockdown and overexpression of miR-199a-5p affected the autophagy regulators

**Fig. 5:**
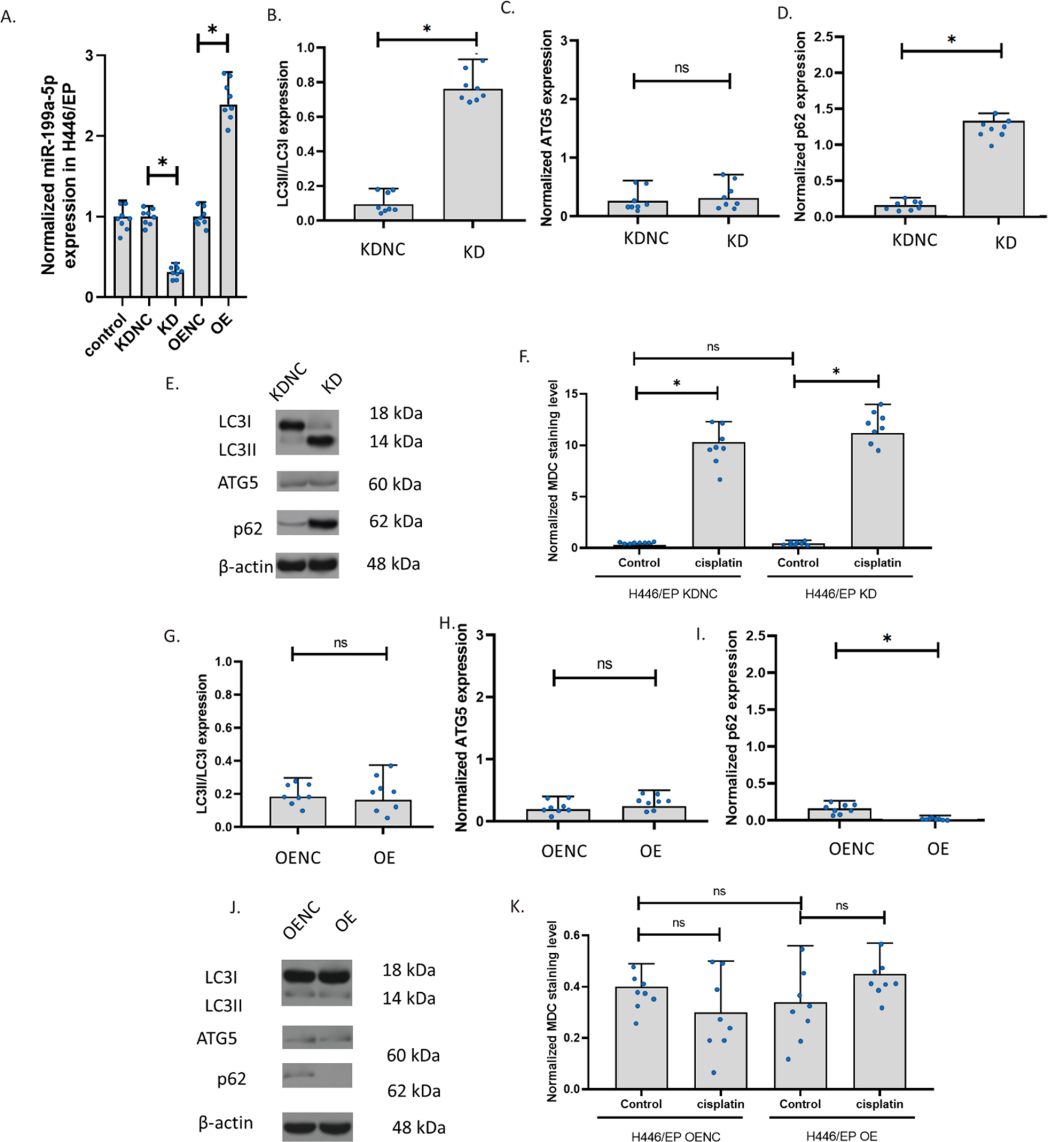
The knockdown and overexpression of miR-199a-5p in H446/EP. **A** The effect of cisplatin and β-elemene on miR-199a-5p expression in H446/EP with knockdown or overexpression of miR-199a-5p. MiR-199a-5p expression was determined using the QPCR assay. Overexpression (OE); knockdown (KD); overexpression negative control; knockdown negative control (KDNC). **B**–**D** LC3II/LC3I, ATG5, and p62 expressions in H446/EP with knockdown of miR-199a-5p. **E** Representative images of **B**–**D**. The expression was measured using the western blotting assay. **F** The MDC staining of autophagolysosomes in cells with drug exposure. The fluorescence images of autophagolysosomes were captured after the MDC staining of cells. **G**–**I** LC3II/LC3I, ATG5, and p62 expressions in H446/EP with overexpression of miR-199a-5p. **J** Representative images of **B**–**D**. **K** The MDC staining of autophagolysosomes in cells with drug exposure. (*p < 0.01)

To explore the role of miR-199a-5p, we knocked down (KD) and overexpressed (OE) miR-199a-5p in H446/EP. In H466/EP, the knockdown decreased miR-199a-5p and the overexpression significantly increased miR-199a-5p (Fig. 5A). We determined the ratio of LC3II and LC3I expression, the level of ATG5, and the levels of p62 to measure the autophagy in H446/EP cells. Results showed that miR-199a-5p knockdown increased LC3II/LC3I and p62 in H446/EP cells, but ATG5 was not affected (Fig. 5B–E). We also observed autophagy in the cells. The staining of the autophagolysosomes with MDC in H446 cells showed that the autophagolysosomes were significantly increased in H446/EP with the miR-199a-5p knockdown. The autophagolysosome signals in the knockdown control with or without cisplatin were all low. The autophagolysosome signals in H446/EP with miR-199a-5p knockdown were not significantly increased after 24-h exposure to cisplatin. We suggested that H446/EP had developed mechanisms that prevent autophagy induced by cisplatin. These results also further confirmed that the miR-199a-5p decreased p62. To further explore the role of miR-199a-5p in H446/EP, we overexpressed miR-199a-5p in H446/EP cells. Results revealed that, after miR-199a-5p overexpression, H446/EP showed a similar ratio of LC3II/LC3I and a similar level of ATG5. However, the expression of p62 was almost completely blocked (Fig. 5G–J). The staining of the autophagolysosomes with MDC in H446/EP cells showed that both the control and the miR-199a-5p overexpression H446/EP had very low signals of autophagolysosomes, the 24-h cisplatin exposure failed to make any changes in the signals of autophagolysosomes (Fig. 5K). These data suggested that the effects of miR-199a-5p on H446 cells were eliminated by the long-time exposure selection of cisplatin.

### The knockdown and overexpression of miR-199a-5p in H69PR

**Fig. 6:**
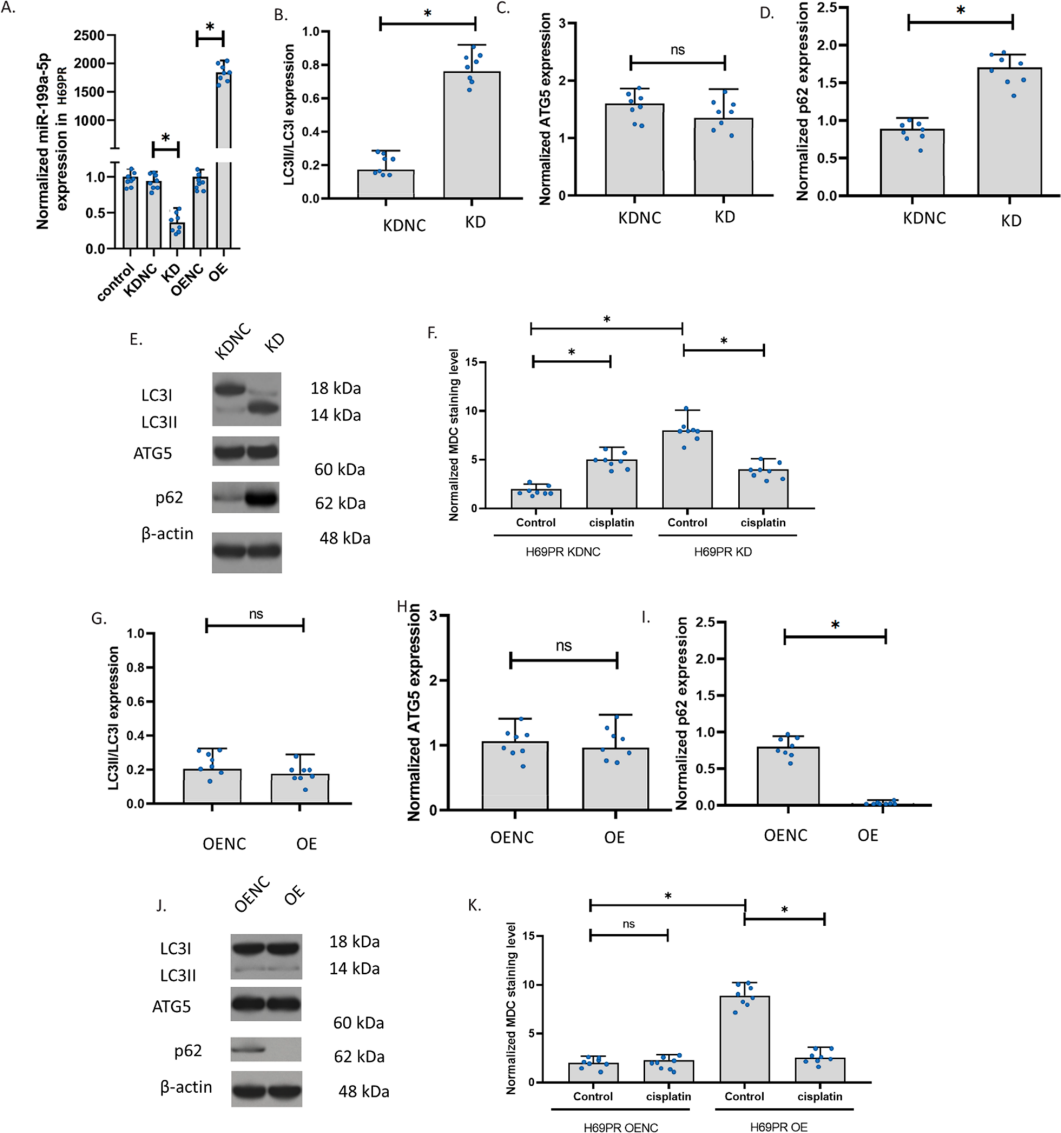
The knockdown and overexpression of miR-199a-5p in H69PR. **A** The effect of cisplatin and β-elemene on miR-199a-5p expression in H69PR with knockdown or overexpression of miR-199a-5p. MiR-199a-5p expression was determined using the QPCR assay. Overexpression (OE); knockdown (KD); overexpression negative control; knockdown negative control (KDNC). **B**–**D** LC3II/LC3I, ATG5, and p62 expressions in H69PR with knockdown of miR-199a-5p. **E** Representative images of **B**–**D**. The expression was measured using the western blotting assay. **F** Image of autophagolysosomes in cells with drug exposure. The fluorescence images of autophagolysosomes were captured after the MDC staining of cells. **G**–**I** LC3II/LC3I, ATG5, and p62 expressions in H69PR with overexpression of miR-199a-5p. **J** Representative images of **B**–**D**. **K** The MDC staining of autophagolysosomes in cells with drug exposure. (*p < 0.01)

To explore the role of miR-199a-5p, we knocked down (KD) and overexpressed (OE) miR-199a-5p in H69PR. In H466, the knockdown slightly decreased miR-199a-5p but the overexpression increased miR-199a-5p up to 1500 folds (Fig. 6A). We determined the ratio of LC3II and LC3I expression, the level of ATG5, and the levels of p62 to measure the autophagy in H69PR cells. Results showed that miR-199a-5p knockdown increased LC3II/LC3I and p62 in H69PR cells, but ATG5 was not affected (Fig. 6B–E). We also observed autophagy in the cells. The staining of the autophagolysosomes with MDC in H69PR cells showed that the autophagolysosomes were significantly increased in H69PR with the miR-199a-5p knockdown. The autophagolysosomes were increased only in the knockdown control but not the H69PR with miR-199a-5p knockdown after 24-h exposure to cisplatin. These results indicated that the miR-199a-5p might decrease p62 and the transformation of LC3I to LC3II and the decrease in miR-199a-5p can result in the insensitivity of H69PR cells to cisplatin. To further explore the role of miR-199a-5p in H69PR, we overexpressed miR-199a-5p in H69PR cells. Results revealed that, after miR-199a-5p overexpression, H69PR showed a similar ratio of LC3II/LC3I and a similar level of ATG5. However, the expression of p62 was remarkably reduced (Fig. 6G–J). The staining of the autophagolysosomes with MDC in H69PR cells showed that both the control and the miR-199a-5p overexpression H69PR had very low signals of autophagolysosomes, the 24-h cisplatin exposure increased the signals of autophagolysosomes in control only (Fig. 6K). These results indicated that overexpression of miR-199a-5p also unsensitized the H69PR to cisplatin.

### Effect of cisplatin on an orthotopic H446 resistance mouse model of SCLC

**Fig. 7:**
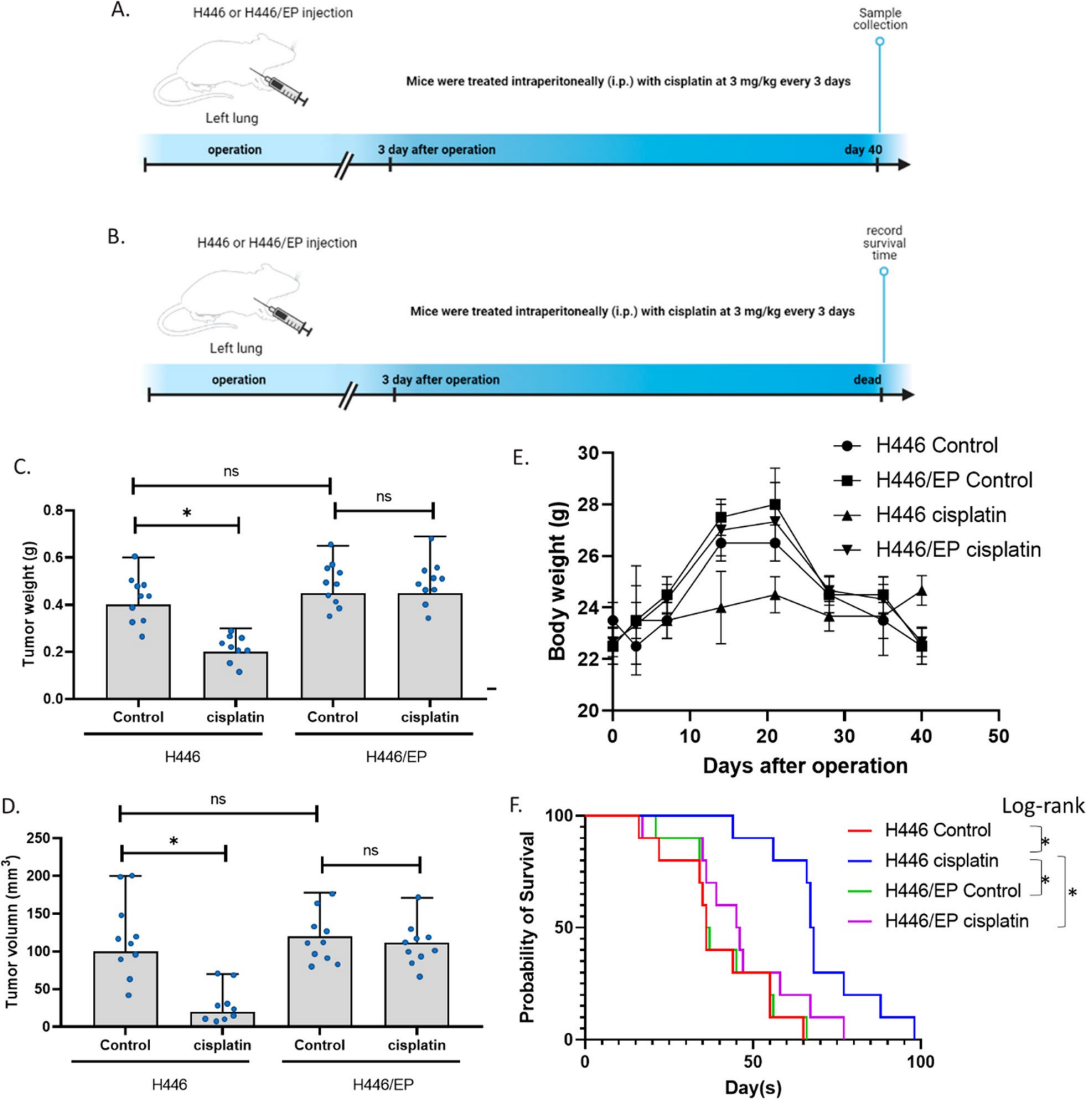
Effect of cisplatin on an orthotopic H446 resistance mouse model of SCLC. **A** Experimental setting of the sample group. **B** Experimental setting of the survival group. **C** Tumor weight of the sample group. **D** Tumor volume of the sample group. **E** Body weight of the sample group. **F** Survival of the survival group. (*p < 0.01)

To further validate the conclusion of the in vivo experiment, we established an orthotopic mouse model of SCLC using H446 and H446/EP. Half of the animals were killed on day 40 for sample collection and the other animals were treated until the death for recording the survival time (Fig. 7A, B). Results showed that at day 40, the cisplatin treatment significantly reduced the tumor weight and volume in H446 tumor animals, however, the cisplatin treatment failed to decrease the tumor weight and volume in H446/EP tumor animals. In addition, the bodyweight of H446 control, H446/EP control, and H446/EP cisplatin groups showed a similar trend: increased before day 20 and decreased after that, while the bodyweight of the H446 cisplatin group generally had no alteration. We suggested that body weight increase of H446 control, H446/EP control, and H446/EP cisplatin groups resulted from the growth of the tumor pathological changes, such as pulmonary effusion, while the decrease after day 20 resulted from the decrease in the intake of food caused by the discomfort from the tumor. Yet, in the H446 cisplatin group, the tumor grow slowly and the body weight was not changed overtimes. In the survival groups, we found that animals in the H446 cisplatin group survival significantly longer than the other three groups. These data suggested that the resistant orthotopic H446 resistance mouse model of SCLC was successful.

### Effect of cisplatin on an orthotopic H446 resistance mouse model of SCLC

**Fig. 8:**
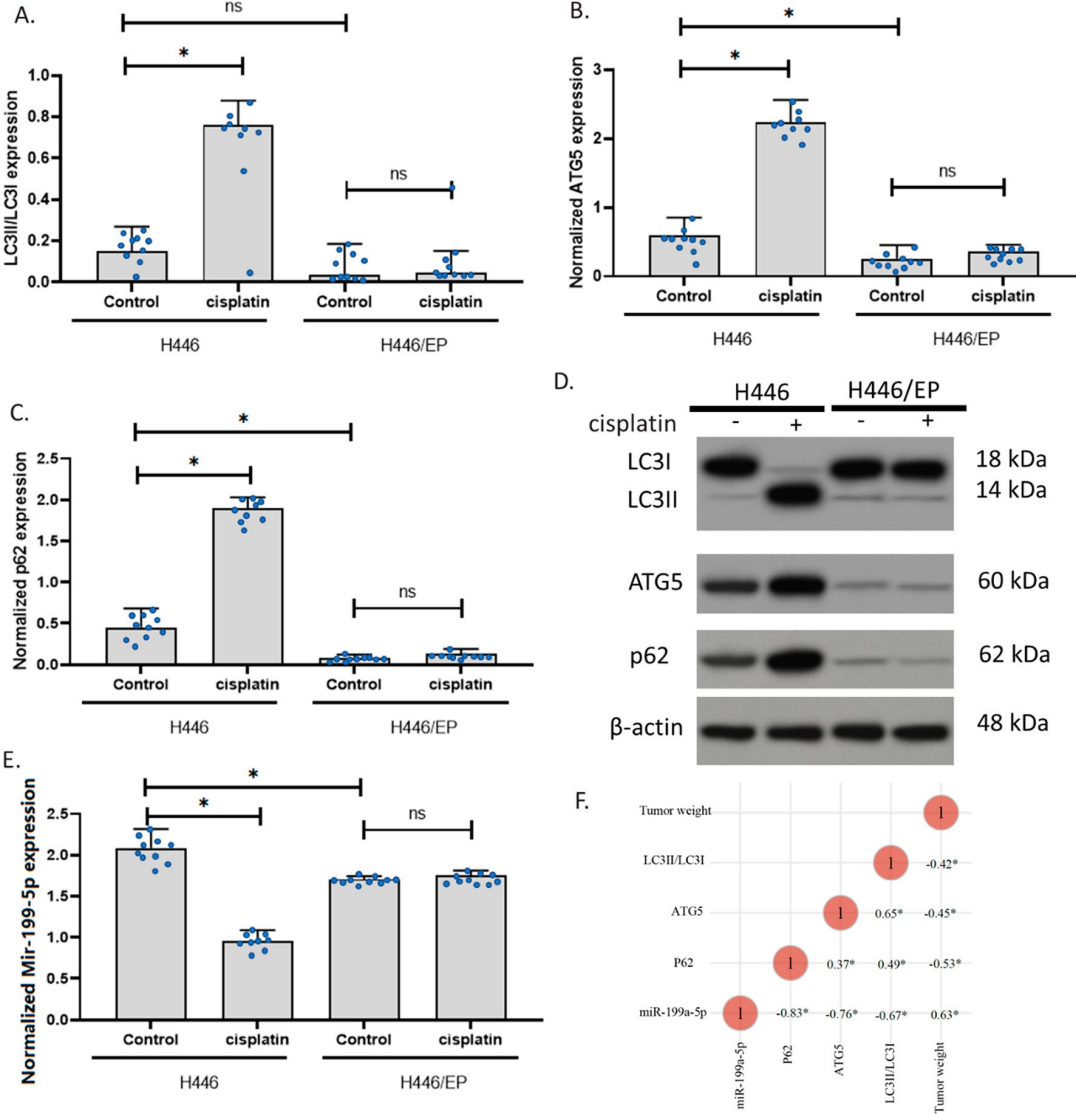
The expression of p62 and miR-199a-5p in mice SCLC samples. **A**–**C** LC3II/LC3I, ATG5, and p62 expressions in H446 and H446/EP model of SCLC with or without cisplatin treatment. The expression was measured using the western blotting assay. **D** Representative images of **A**–**C**. **E** Expression of mir-199-5p expressions in H446 and H446/EP model of SCLC with or without cisplatin treatment. **F** Correlation of the data of all collected tumor samples. (*p < 0.01)

We further determined the expression of LC3II/LC3I, ATG5, p62, and miR-199a-5p in the samples we collected. Results showed that cisplatin increased LC3II/LC3I, ATG5, and p62 in the H446 mouse, but not in H446/EP mouse. These results were similar to the results in cell experiments. This indicated that the drug resistance of cisplatin potentially resulted from the insensitivity of autophagy induction in the mouse. In addition, compared to the H446 tumor, H446/EP had a similar ratio of LC3II/LC3I, but a significantly lower level of ATG5 and p62. These results were also consistent with the cell experiments, suggesting that the cells kept their autophagy level in vivo as in vitro. therefore, these data validated that H446 might develop autophagy-associated resistant mechanisms in vivo (Fig. 8A–D). Additionally, miR-199a-5p expression was decreased by cisplatin treatment in H446 mouse only, indicating that it is potentially associated. Thus, we analyzed the correlation of tumor weight, LC3II/LC3I, ATG5, p62, and miR-199a-5p in all the samples we collected. Results showed that miR-199a-5p was negatively associated with LC3II/LC3I, ATG5, and p62 and positively correlated with the tumor weight. These data indicated that miR-199a-5p might be a beneficial molecule that negatively regulates autophagy. Strikingly, miR-199a-5p is strongly correlated with p62 level with a coefficient of − 0.83, which further supported that miR-199a-5p had direct interaction with p62 protein (Fig. 8F).

## Discussion

Small cell lung cancer is a type of highly aggressive lung cancer. As it typically causes symptoms in early-stage patients, it can be discovered at earlier stages, therefore, chemotherapy drugs are usually implemented almost throughout the entire course of treatment, causing more chance for the cells to develop MDR [37]. Clinical cancer treatment involves many drugs that might potentially make a difference such as anesthetics [38–41]. The doses of chemotherapy are critical for the treatment of SCLC. In this study, we conducted a series of CCK-8 assays to determine the effective doses of five commonly used chemo agents including cisplatin, etoposide, paclitexal, epirubicin, and irinotecan with H446 and H446/EP. The CCK-8 assay was a simple-step cell viability assay with stable results [42] and has fewer steps than the MTT assay [31, 40, 43]. Thus CCK-8 was conducive for our serious viability assay. The evaluation of IC50 and DRIC revealed that H446/EP showed MDR property. In this study, we studied one of the typical chemotherapy drugs cisplatin using the H446/EP model. H446/EP is a wildly used resistance cell line that was reported a lot previously [29, 44]. On one hand, this enables us to generate the cell line stably. On the other, this makes our results easier to compare with others’ results. In addition, in the clinical cancer treatment, patients who develop multi-drug resistance are a much more difficult situation than specific cisplatin-resistant, if multi-drug resistance has a different mechanism compared to specific cisplatin-resistant, it is more valuable to study multi-drug resistance. When eliminating multi-drug resistance, it does not necessarily need to eliminate the resistance of cancer to all drugs, improving the sensitivity of cancer to one drug would be beneficial. Therefore, as for a higher clinical value, we study the cisplatin-resistant in multi-drug resistance model.

Many previous studies have reported that cisplatin can induce MDR in H446 cells through multiple pathways [45, 46]. Our results demonstrated that the drug resistance to cisplatin was resulted (at least partly) from the insensitivity of autophagy induction. Accumulating literature reported that abnormal autophagy plays a critical role in cancer MDR development [47]. Nevertheless, to date, few researchers are studying the inhibition of autophagy in drug-resistant lung cancer cells. In the present study, we demonstrated that autophagy was involved in H446/EP, and the potential mechanism included the activation of LC3I/LC3II conversion, ATG5 expression, and p62 expression. LC3II conversed from LC3I has been wildly accepted to be associated with the movement of mature autophagosomes along microtubular tracks [48], while ATG5 has been one of the indicators for autophagy and it plays essential roles in the elongation and expansion of phagophore membrane. The downregulation of ATG5 could prevent the autophagosome from maturation and thereby block autophagy [49]. The p62 protein, also named SQSTM1, is involved in various signaling pathways and cellular functions including autophagy [50]. The p62 is a multifunctional protein in autophagy. The binding of p62 directly to LC3 via a short LC3 interaction region is one of the most critical mechanisms to deliver selective autophagic cargo for the bioprocess. The p62 overexpression increases the aggregation of ubiquitinated proteins and has a protective effect on cell survival, while p62 decrease results in some diseases by damaging autophagic degradation. However, the p62 protein itself is degraded by autophagy and can accumulate when the autophagy level is reduced. Therefore, on one hand, p62 is upstream of autophagy. On the other hand, p62 is degraded by autophagy. Normally, the accumulation of p62 means decreased activity of autophagy, but in our study, we also observed the other two indicators for autophagy: ATG5 and LC3. Increased level of ATG5 and LC3II means an increase in autophagy. Under the effect of cisplatin, ATG5 and LC3 indicated that the autophagy was increased, but in our results, the p62 was also increased. We suggested that this was because the cisplatin remarkably up-regulated p62 but did not affect ATG5 and LC3II. Only p62 contributed to the increase in autophagy, but not ATG5 and LC3. Thus, the remarkably up-regulated p62 was not degraded fast enough by autophagy, and hence p62 accumulated. In addition, the drug resistance triggered the miR-199a-5p/p62 axis. The p62 in resistance cells was very low, therefore, cells became insensitive to cisplatin.

Growing lines of evidence supported the abnormal expression of miR-199a-5p in MDR cell lines. A study showed that cisplatin-induced the decrease of miR-199a-5p expression in human osteosarcoma cells MG63 [21]. Another study reported that the expression of miR-199a-5p in leukemia cells from relapsed/refractory patients was lower than that from patients with complete remission [27]. However, our results showed that H446/EP expressed a higher level of miR-199a-5p. But the miR-199a-5p expression was not induced by short-time exposure of cisplatin. We suggested that the reaction of miR-199a-5p expression to cisplatin was cancer-type specific. In the KD experiment of H446, KD was not strong, we think it might be because the level of miR-199a-5p in H446 was already low before KD and can not be further decreased. On the other hand, in H446/EP, KD reduced miR-199a-5p by 70%, we think this was because in H446/EP was higher than in H446, thus miR-199a-5p can be decreased. Another striking finding of this study was that miR-199a-5p could directly bind to p62 mRNA resulting in the degradation of p62 in autophagy repressive H446/EP cells. The regulation of p62-mediated autophagy by miR-199a-5p was found to be a potential mechanism of small cell lung cancer cisplatin resistance. In addition, the ATG5 protein was also critical in the regulations of autophagy. Although ATG5 was involved in the MDR of H446, our data suggested that it is not affected by miR-199a-5p.

Our study was the first paper that reported an abnormally high expression of miR-199a-5p in drug resistance lung cancer cells. This study is conducive to the development of miR-199a-5p as a potential biomarker for the occurrence of drug resistance in lung cancer cells. Our data suggested that miR-199a-5p could be a pharmacological target for p62 protein and it was critical in mediating autophagy regulation by cisplatin. In addition, sodium homeostasis has been suggested to be involved in autophagy, but whether the role of sodium channels in cancer [51] is associated with autophagy, cisplatin, and MDR needs further investment. Many studies identified cancer biomarkers [52]. We think miR-199a-5p can also be a potential cancer biomarker. In addition, so far, it is still controversial that autophagy is a protective or responsive mechanism upon each treatment, the same thing here for cisplatin on small cell lung cancer. It is important to link autophagy degrees with cell growth rate, apoptosis et al. or, it could very much be possible, autophagy is just an independent event that has nothing to do with cell growth rate and apoptosis. However, we think autophagy is not the only mechanism of drug resistance. As we do not want to over-interpret the results, our data only suggested that autophagy might be associated with cell viability and we are sure that autophagy is altered during the resistance.

Another aspect that can be further explored is the potential regulation of miR-199a-5p by natural drugs. In recent years, many naturally occurring compounds have been studied and implemented in the clinical therapy of human disease [53–57]. Accumulating evidence suggests that chemotherapy supplied by traditional medicine can achieve desirable outcomes in clinical cancer treatment, including higher efficiency and lower side effects [58, 59]. For example, the natural compound β-elemene has been shown to induce autophagy in cancer cells [60], at the same time, it can suppress the multidrug-resistant cell lines [61]. We believe that autophagy is one of the mechanisms of these multidrug-resistant effects. A better understanding of autophagy in multidrug-resistant cancer cells can provide evidence for the use of some of these pharmacological active compounds.

## Conclusions

The regulation of p62-mediated autophagy by miR-199a-5p was a potential mechanism of SCLC cisplatin resistance.

## Supplementary Information

**Additional file 1: Table S1. **DRIC of multiple drugs in H446 (CCK). **Table S2.** DRIC of multiple drugs in H446 (LDH).

## Data Availability

The raw data of this study are provided from the corresponding author with a reasonable request.

## Abbreviations

OE: Overexpressed
KD: Knocked down
NSCLC: Non-small cell lung cancers
SCLC: Small cell lung cancer
MDR: Multidrug resistance
OENC: Overexpressed negative control
KDNC: Knockdown negative control
WT: Wild-type
MDC: Monodansylcadaverine

## References

[1] Skřičková J, Kadlec B, Venclíček O, Merta Z (2018). Lung cancer. Casopis lekaru ceskych.

[2] Bade BC, Dela Cruz CS (2020). Lung cancer 2020: epidemiology, etiology, and prevention. Clin Chest Med.

[3] Collins LG, Haines C, Perkel R, Enck RE (2007). Lung cancer: diagnosis and management. Am Fam Physician.

[4] Saltos A, Shafique M, Chiappori A (2020). Update on the biology, management, and treatment of small cell lung cancer (SCLC). Front Oncol.

[5] Zahreddine H, Borden KL (2013). Mechanisms and insights into drug resistance in cancer. Front Pharmacol.

[6] Bukowski K, Kciuk M, Kontek R (2020). Mechanisms of multidrug resistance in cancer chemotherapy. Int J Mol Sci.

[7] Wu Q, Yang Z, Nie Y, Shi Y, Fan D (2014). Multi-drug resistance in cancer chemotherapeutics: mechanisms and lab approaches. Cancer Lett.

[8] Li X, He S, Ma B (2020). Autophagy and autophagy-related proteins in cancer. Mol Cancer.

[9] Xu Z, Jiang H, Zhu Y, Wang H, Jiang J, Chen L, Xu W, Hu T, Cho CH (2017). Cryptotanshinone induces ROS-dependent autophagy in multidrug-resistant colon cancer cells. Chem Biol Interact.

[10] Zhang X, Chen X, Guo Y, Jia HR, Jiang YW, Wu FG (2020). Endosome/lysosome-detained supramolecular nanogels as an efflux retarder and autophagy inhibitor for repeated photodynamic therapy of multidrug-resistant cancer. Nanoscale Horiz.

[11] Utaipan T, Athipornchai A, Suksamrarn A, Chunsrivirot S, Chunglok W (2017). Isomahanine induces endoplasmic reticulum stress and simultaneously triggers p38 MAPK-mediated apoptosis and autophagy in multidrug-resistant human oral squamous cell carcinoma cells. Oncol Rep.

[12] Mizushima N, Levine B, Cuervo AM, Klionsky DJ (2008). Autophagy fights disease through cellular self-digestion. Nature.

[13] Feng Y, He D, Yao Z, Klionsky DJ (2014). The machinery of macroautophagy. Cell Res.

[14] Mishima Y, Terui Y, Mishima Y, Taniyama A, Kuniyoshi R, Takizawa T, Kimura S, Ozawa K, Hatake K (2008). Autophagy and autophagic cell death are next targets for elimination of the resistance to tyrosine kinase inhibitors. Cancer Sci.

[15] Hu YL, Jahangiri A, Delay M, Aghi MK (2012). Tumor cell autophagy as an adaptive response mediating resistance to treatments such as antiangiogenic therapy. Cancer Res.

[16] Tanida I, Ueno T, Kominami E (2008). LC3 and autophagy. Methods Mol Biol (Clifton, N.J.).

[17] Komatsu M, Kageyama S, Ichimura Y (2012). p62/SQSTM1/A170: physiology and pathology. Pharmacol Res.

[18] Komatsu M, Waguri S, Koike M, Sou Y-S, Ueno T, Hara T, Mizushima N, Iwata J-I, Ezaki J, Murata S (2007). Homeostatic levels of p62 control cytoplasmic inclusion body formation in autophagy-deficient mice. Cell.

[19] Pankiv S, Clausen TH, Lamark T, Brech A, Bruun J-A, Outzen H, Øvervatn A, Bjørkøy G, Johansen T (2007). p62/SQSTM1 binds directly to Atg8/LC3 to facilitate degradation of ubiquitinated protein aggregates by autophagy*. J Biol Chem.

[20] Liu WJ, Ye L, Huang WF, Guo LJ, Xu ZG, Wu HL, Yang C, Liu HF (2016). p62 links the autophagy pathway and the ubiqutin–proteasome system upon ubiquitinated protein degradation. Cell Mol Biol Lett.

[21] Li Y, Jiang W, Hu Y, Da Z, Zeng C, Tu M, Deng Z, Xiao W (2016). MicroRNA-199a-5p inhibits cisplatin-induced drug resistance via inhibition of autophagy in osteosarcoma cells. Oncol Lett.

[22] Ahmadi A, Khansarinejad B, Hosseinkhani S, Ghanei M, Mowla SJ (2017). miR-199a-5p and miR-495 target GRP78 within UPR pathway of lung cancer. Gene.

[23] Hua Q, Jin M, Mi B, Xu F, Li T, Zhao L, Liu J, Huang G (2019). LINC01123, a c-Myc-activated long non-coding RNA, promotes proliferation and aerobic glycolysis of non-small cell lung cancer through miR-199a-5p/c-Myc axis. J Hematol Oncol.

[24] Li Y, Wang D, Li X, Shao Y, He Y, Yu H, Ma Z (2019). MiR-199a-5p suppresses non-small cell lung cancer via targeting MAP3K11. J Cancer.

[25] Li DJ, Wang X, Yin WH, Niu K, Zhu W, Fang N (2020). MiR-199a-5p suppresses proliferation and invasion of human laryngeal cancer cells. Eur Rev Med Pharmacol Sci.

[26] Zhu QD, Zhou QQ, Dong L, Huang Z, Wu F, Deng X (2018). MiR-199a-5p inhibits the growth and metastasis of colorectal cancer cells by targeting ROCK1. Technol Cancer Res Treat.

[27] Li Y, Zhang G, Wu B, Yang W, Liu Z (2019). miR-199a-5p represses protective autophagy and overcomes chemoresistance by directly targeting DRAM1 in acute myeloid leukemia. J Oncol.

[28] Chen PH, Liu AJ, Ho KH, Chiu YT, Anne Lin ZH, Lee YT, Shih CM, Chen KC (2018). MicroRNA-199a/b-5p enhance imatinib efficacy via repressing WNT2 signaling-mediated protective autophagy in imatinib-resistant chronic myeloid leukemia cells. Chem Biol Interact.

[29] Pan B, Chen Y, Song H, Xu Y, Wang R, Chen L (2015). Mir-24-3p downregulation contributes to VP16-DDP resistance in small-cell lung cancer by targeting ATG4A. Oncotarget.

[30] Wu Z, Ou L, Wang C, Yang L, Wang P, Liu H, Xiong Y, Sun K, Zhang R, Zhu X (2017). Icaritin induces MC3T3-E1 subclone14 cell differentiation through estrogen receptor-mediated ERK1/2 and p38 signaling activation. Biomed Pharmacother.

[31] Liu H, Dilger JP, Lin J (2020). The role of transient receptor potential melastatin 7 (TRPM7) in cell viability: a potential target to suppress breast cancer cell cycle. Cancers.

[32] Liu X, Liu H, Xiong Y, Yang L, Wang C, Zhang R, Zhu X (2018). Postmenopausal osteoporosis is associated with the regulation of SP, CGRP, VIP, and NPY. Biomed Pharmacother.

[33] Xue JF, Shi ZM, Zou J, Li XL (2017). Inhibition of PI3K/AKT/mTOR signaling pathway promotes autophagy of articular chondrocytes and attenuates inflammatory response in rats with osteoarthritis. Biomed Pharmacother.

[34] Li X, Peng B, Zhu X, Wang P, Xiong Y, Liu H, Sun K, Wang H, Ou L, Wu Z (2017). Changes in related circular RNAs following ERbeta knockdown and the relationship to rBMSC osteogenesis. Biochem Biophys Res Commun.

[35] Clément T, Salone V, Rederstorff M (2015). Dual luciferase gene reporter assays to study miRNA function. Methods Mol Biol (Clifton, N.J.).

[36] Isobe T, Onn A, Morgensztern D, Jacoby JJ, Wu W, Shintani T, Itasaka S, Shibuya K, Koo PJ, O'Reilly MS (2013). Evaluation of novel orthotopic nude mouse models for human small-cell lung cancer. J Thorac Oncol.

[37] Byers LA, Rudin CM (2015). Small cell lung cancer: where do we go from here?. Cancer.

[38] Li R, Liu H, Dilger JP, Lin J (2018). Effect of Propofol on breast Cancer cell, the immune system, and patient outcome. BMC Anesthesiol.

[39] Li R, Huang Y, Liu H, Dilger JP, Lin J. Comparing volatile and intravenous anesthetics in a mouse model of breast cancer metastasis. p. 2162.

[40] Liu H (2020). A prospective for the potential effect of local anesthetics on stem-like cells in colon cancer. Biomed J Sci Tech Res.

[41] Liu H (2020). A clinical mini-review: clinical use of local anesthetics in cancer surgeries. Gazette Med Sci.

[42] Liu H, Dilger JP, Lin J (2020). Effects of local anesthetics on cancer cells. Pharmacol Ther.

[43] Li R, Xiao C, Liu H, Huang Y, Dilger JP, Lin J (2018). Effects of local anesthetics on breast cancer cell viability and migration. BMC Cancer.

[44] Chen Y, Yang X, Xu Y, Cao J, Chen L (2017). Genomic analysis of drug resistant small cell lung cancer cell lines by combining mRNA and miRNA expression profiling. Oncol Lett.

[45] Li W, Shi Y, Wang R, Pan L, Ma L, Jin F (2018). Resveratrol promotes the sensitivity of small-cell lung cancer H446 cells to cisplatin by regulating intrinsic apoptosis. Int J Oncol.

[46] Liu HN, Qie P, Yang G, Song YB (2018). miR-181b inhibits chemoresistance in cisplatin-resistant H446 small cell lung cancer cells by targeting Bcl-2. Arch Med Sci.

[47] Li YJ, Lei YH, Yao N, Wang CR, Hu N, Ye WC, Zhang DM, Chen ZS (2017). Autophagy and multidrug resistance in cancer. Chin J Cancer.

[48] Xie R, Nguyen S, McKeehan WL, Liu L (2010). Acetylated microtubules are required for fusion of autophagosomes with lysosomes. BMC Cell Biol.

[49] Arakawa S, Honda S, Yamaguchi H, Shimizu S (2017). Molecular mechanisms and physiological roles of Atg5/Atg7-independent alternative autophagy. Proc Jpn Acad Ser B Phys Biol Sci.

[50] Tao M, Liu T, You Q, Jiang Z (2020). p62 as a therapeutic target for tumor. Eur J Med Chem.

[51] Liu H (2020). Nav channels in cancers: nonclassical roles. Glob J Cancer Ther.

[52] Li Y, Liu H, Han Y. Potential roles of cornichon family AMPA receptor auxiliary protein 4 (CNIH4) in head and neck squamous cell carcinoma. 2021. 10.3233/CBM-220143 PMC12364253 36404537

[53] Chen G, Wang C, Wang J, Yin S, Gao H, Xiang LU, Liu H, Xiong Y, Wang P, Zhu X (2016). Antiosteoporotic effect of icariin in ovariectomized rats is mediated via the Wnt/beta-catenin pathway. Exp Ther Med.

[54] Liu H, Xiong Y, Wang H, Yang L, Wang C, Liu X, Wu Z, Li X, Ou L, Zhang R (2018). Effects of water extract from epimedium on neuropeptide signaling in an ovariectomized osteoporosis rat model. J Ethnopharmacol.

[55] Liu H, Xiong Y, Zhu X, Gao H, Yin S, Wang J, Chen G, Wang C, Xiang L, Wang P (2017). Icariin improves osteoporosis, inhibits the expression of PPARgamma, C/EBPalpha, FABP4 mRNA, N1ICD and jagged1 proteins, and increases Notch2 mRNA in ovariectomized rats. Exp Ther Med.

[56] Haixia W, Shu M, Li Y, Panpan W, Kehuan S, Yingquan X, Hengrui L, Xiaoguang L, Zhidi W, Ling O (2020). Effectiveness associated with different therapies for senile osteopo-rosis: a network meta-analysis. J Tradit Chin Med.

[57] Wang C, Chen G, Wang J, Liu H, Xiong Y, Wang P, Yang L, Zhu X, Zhang R (2016). Effect of herba epimedium extract on bone mineral density and microstructure in ovariectomised rat. J Pharm Biomed Sci.

[58] Liu H (2020). Effect of traditional medicine on clinical cancer. Biomed J Sci Tech Res.

[59] Xiang Y, Guo Z, Zhu P, Chen J, Huang Y (2019). Traditional Chinese medicine as a cancer treatment: modern perspectives of ancient but advanced science. Cancer Med.

[60] Guan C, Liu W, Yue Y, Jin H, Wang X, Wang XJ (2014). Inhibitory effect of β-elemene on human breast cancer cells. Int J Clin Exp Pathol.

[61] Deng M, Liu B, Song H, Yu R, Zou D, Chen Y, Ma Y, Lv F, Xu L, Zhang Z (2020). β-Elemene inhibits the metastasis of multidrug-resistant gastric cancer cells through miR-1323/Cbl-b/EGFR pathway. Phytomed Int J Phytother Phytopharmacol.

[62] Liu H, Dilger JP, Lin J (2021). Lidocaine suppresses viability and migration of human breast cancer cells: TRPM7 as a target for some breast cancer cell lines. Cancers.

