# Supplementary material for: The regulation of autophagy by the miR-199a-5p/p62 axis was a potential mechanism of small cell lung cancer cisplatin resistance
Source: Cancer Cell Int. 2022 Mar 15;22:120. doi: 10.1186/s12935-022-02505-1 (PMC8922820; doi:10.1186/s12935-022-02505-1)
Supplement: Supplementary file 1 — Additional file 1: Table S1. DRIC of multiple drugs in H446 (CCK). Table S2. DRIC of multiple drugs in H446 (LDH). [file 12935_2022_2505_MOESM1_ESM.docx]

**Table S1. DRIC of multiple drugs in H446 (CCK)**

| Drug (μg/ml) | IC_50_ (mean±SD) | | DRIC |
| --- | --- | --- | --- |
|  | H446 | H446/EP |  |
| cisplatin | 0.59±0.33 | 17.67±2.01^*^ | 29.95 |
| etoposide | 0.38±0.19 | 22.21±2.12^*^ | 58.45 |
| paclitexal | 0.14±0.09 | 0.78±0.89^*^ | 5.57 |
| epirubicin | 0.61±0.22 | 5.98±0.89^*^ | 9.80 |
| irinotecan | 18.98±2.11 | 110.86±4.89^*^ | 5.84 |
| vinorelbine | 4.45±0.98 | 9.32±1.99^*^ | 2.09 |

Note: SD, standard deviation; DRIC, drug resistance indices for cancer; FC, fold change. *p<0.01.

**Table S2. DRIC of multiple drugs in H446 (LDH)**

| Drug (μg/ml) | EC_50_ (mean±SD) | | DRIC |
| --- | --- | --- | --- |
|  | H446 | H446/EP |  |
| cisplatin | 0.63±0.23 | 19.97±2.22^*^ | 31.70 |
| etoposide | 0.45±0.22 | 25.66±4.15^*^ | 57.02 |
| paclitexal | 0.20±0.08 | 1.12±0.29^*^ | 5.60 |
| epirubicin | 0.68±0.12 | 6.32±0.45^*^ | 9.29 |
| irinotecan | 22.18±3.33 | 100.36±3.24^*^ | 4.52 |
| vinorelbine | 5.46±0.45 | 11.92±3.56^*^ | 2.18 |

Note: SD, standard deviation; DRIC, drug resistance indices for cancer; FC, fold change. *p<0.01.
